# Pneumoconiosis in a polytetrafluoroethylene (PTFE) spray worker: a case report with an occupational hygiene study

**DOI:** 10.1186/s40557-018-0248-6

**Published:** 2018-06-04

**Authors:** Namhoon Lee, Kiook Baek, Soohyun Park, Inho Hwang, Insung Chung, Wonil Choi, Hyera Jung, Miyoung Lee, Seonhee Yang

**Affiliations:** 1 Department of Occupational and Environmental Medicine, Keimyung University Dongsan Medical Center, 1095 Dalgubeol-daero, Dalseo-gu, Daegu, 42601 South Korea; 2 Nano Convergence Practical Application Center, Daegu, South Korea; 3 Korean Industrial Health Association, Daegu, South Korea; 4 Department of Preventive Medicine, Keimyung University School of Medicine, Daegu, South Korea; 5 Department of Internal Medicine, Keimyung University School of Medicine, Daegu, South Korea; 6 Department of Pathology, Keimyung University School of Medicine, Daegu, South Korea

**Keywords:** Occupational diseases, Pneumoconiosis, Polytetrafluoroethylene

## Abstract

**Background:**

Using analysis of air samples from the workplace, we report on one case of pneumoconiosis in an individual who has been working in a polytetrafluoroethylene (PTFE) spraying process for 28 years.

**Case presentation:**

The patient was diagnosed with granulomatous lung disease caused by PTFE using computed tomography (CT), lung biopsy and electron microscopy. To assess the qualitative and quantitative exposure to PTFE in workplace, Fourier transform infrared spectroscopy (FT-IR), energy-dispersive X-ray spectroscopy (EDX) and thermogravimetric analysis (TGA) were performed on air samples from the workplace. The presence of PTFE particles was confirmed, and the airborne concentration of PTFE was estimated to be 0.75 mg/m^3^.

**Conclusions:**

This case demonstrates that long-term exposure to PTFE spraying can cause granulomatous lung lesions such as pneumoconiosis; such lesions appear to be caused not by the degradation products of PTFE from high temperatures but by spraying the particles of PTFE. Along with air-sampling analysis, we suggest monitoring the concentration of airborne PTFE particles related to chronic lung disease.

## Background

Fluoropolymers are fluorinated carbon-based polymers with multiple carbon-fluorinated bonds [1]. Fluoropolymers have properties of lubricity, chemical inertness, strength, plasticity, and thermal stability. These materials are widely used in gaskets, coating, self-lubricating bearings, food manufacturing machinery, household products such as nonstick cooking utensils, and other applications [2].

Acute lung toxicity from PTFE fumes and chronic foreign body reactions from injected PTFE have been reported. PTFE degrades at temperatures higher than 360 °C, produces toxic fumes, and causes severe lung injury [3, 4]. Fluorocarbon-containing aerosol product exposure due to spraying can also cause acute lung injury [5–7]. Chronic lung disease found in PTFE-spraying workers has been reported [8]. However, the pathophysiology of PTFE particle-induced chronic lung disease has not been reported. Furthermore, measurement of the airborne concentration of PTFE particles has not been reported.

Here, using analysis of air samples from a workplace, we report one case of small airway-centered granulomatosis pneumonitis after long-term exposure to the PTFE spray-coating process. An exposure assessment was also performed. The present study protocol was reviewed and approved by the institutional review board of Keimyung University Dongsan Medical Center (IRB No. 2016–02–024-005).

## Case presentation

### The case

#### Patient

Male patient aged 46 years at the time of the first visit for diagnosis.

#### Chief complaint

Abnormal chest X-ray during health examination.

#### History of present illness

The patient displayed no symptoms while working in the PTFE spray-coating process for 28 years.

#### Social history

The patient had never smoked or consumed alcohol.

#### Past medical history

The patient had no history of hypertension, diabetes, or tuberculosis.

#### Family history

The family history was unremarkable and noncontributory.

#### Clinical process

**Fig. 1:**
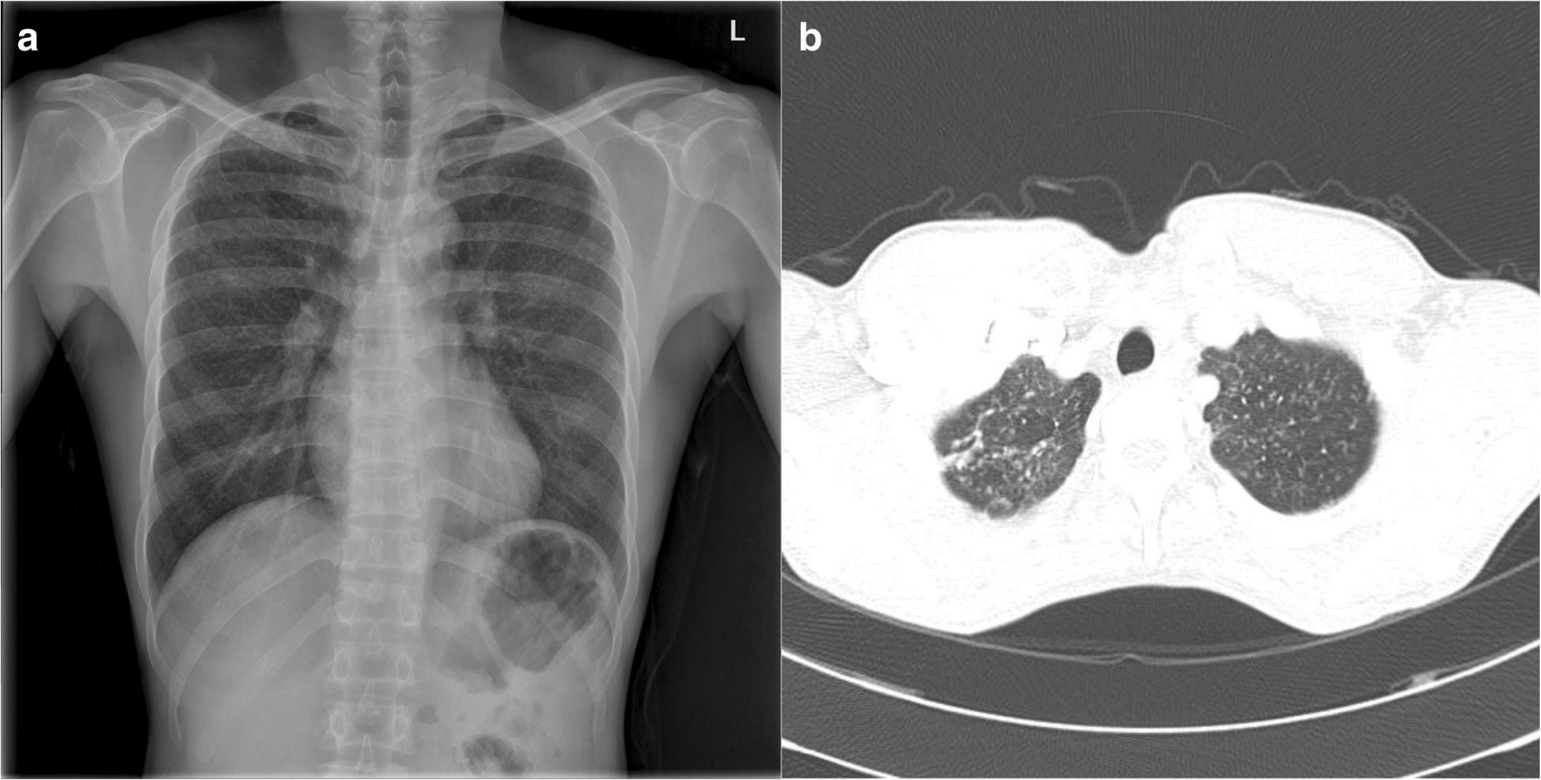
**a** Chest radiograph showing multiple bilateral nodules in both lung fields. **b** CT scan of the chest showing numerous tiny nodules with perilymphatic distribution without lymph-node calcification

**Fig. 2:**
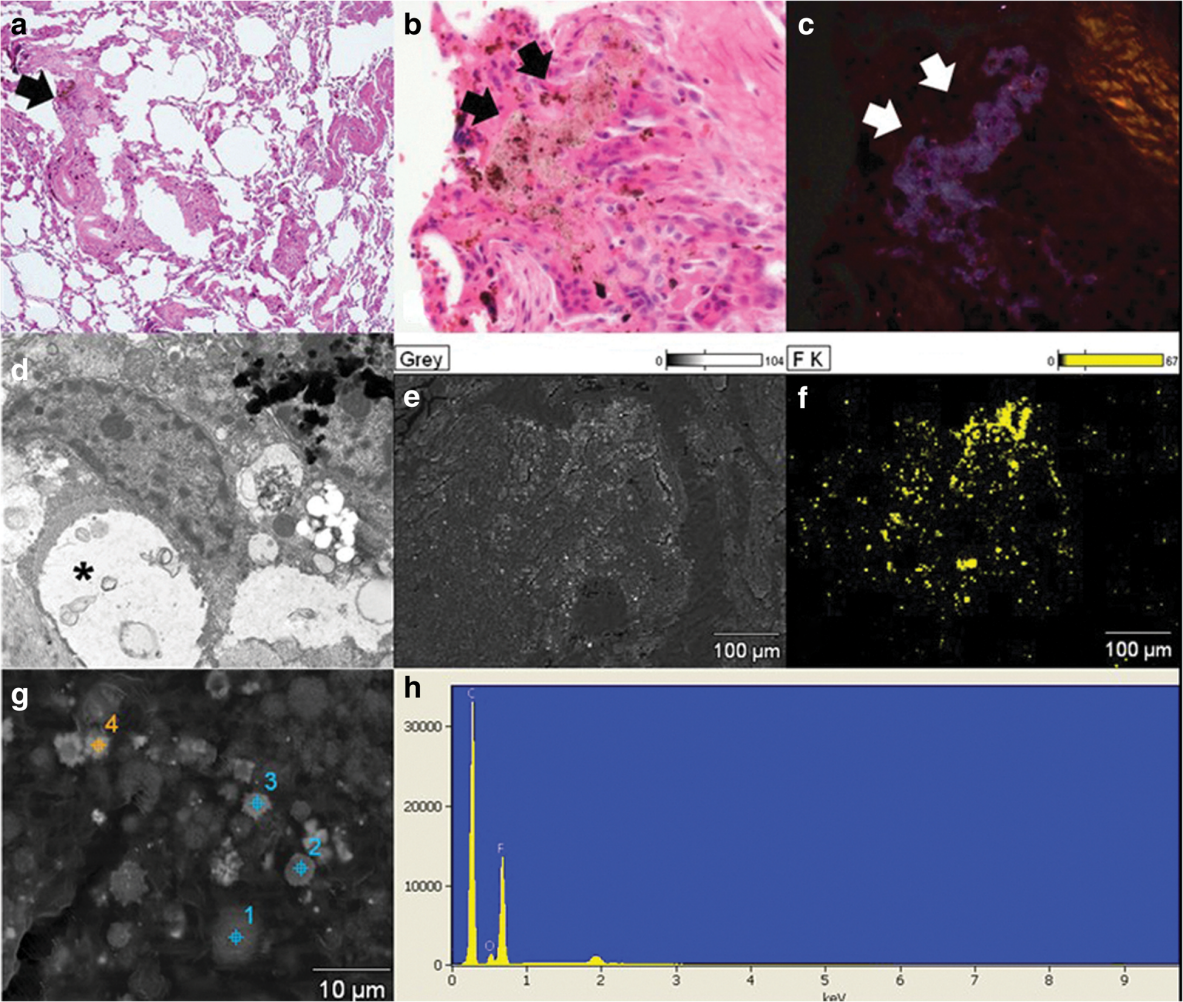
Histologic features of a case of polytetrafluoroethylene (PTFE) exposure-induced pulmonary granulomatous lesions (**a** and **b**). **a** The specimen shows mainly small airway- centered granulomatous micronodular lesions (arrow). **b** Higher magnification of the specimen shows multinucleated giant cells containing amorphous transparent particles (black arrows). **c** Polarizing microscopic finding of the same multinucleated giant cell found in Fig. **b** reveals the birefringent particle (white arrows). **a** to **c** Hematoxylin-Eosin stain (Magnification: A: × 40, B and C: × 400). **d** Transmission electron microscopic feature show intracytoplasmic electron lucent amorphous materials (asterisks) in the histiocyte. **e** and **f** Scanning electron microscopic feature (**e**) and comparing element mapping of fluorine (yellow) reveals the presence of fluorine in the pulmonary lesion. **g** The scanning electron microscopic feature of the lesion revealing fluorine in Fig. **f** shows multiple round to oval granular material measuring 2–6 μm. (Original magnification, **d**: × 3500, **e** and **f**: × 500, **g** × 5000). **h**: Energy-dispersive X-ray spectroscopy (EDS) spectrum of a particle in a round particle (target 1 shown in Fig. **g**) found in multinucleated giant cell showing a prominent peak for fluorine (F) but with other associated elements such as carbon (C), and oxygen (O)

**Fig. 3:**
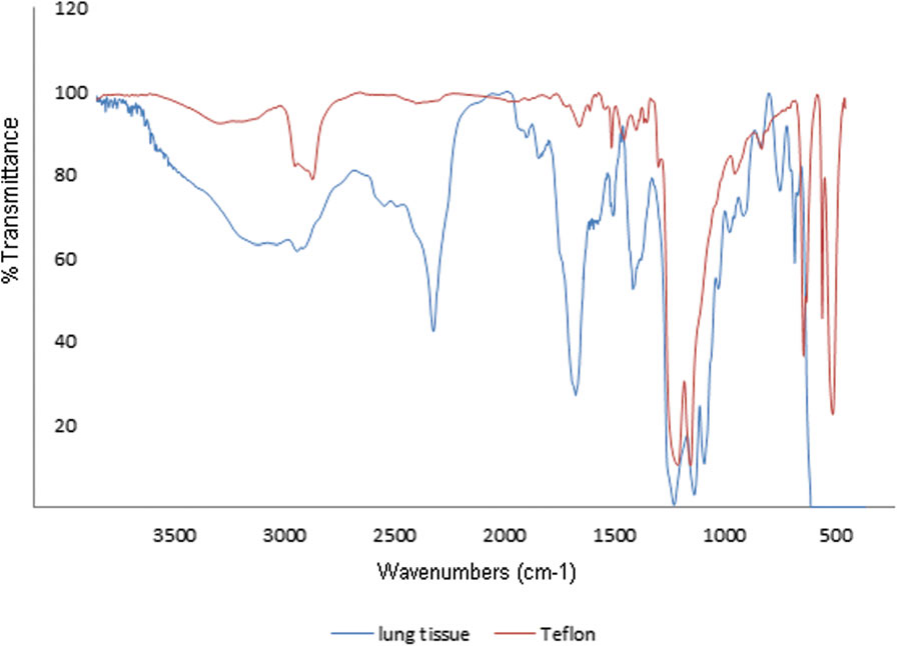
FT-IR analysis of the lung tissue of the patient shows the presence of PTFE compared with the standard PTFE peak result

The patient was diagnosed with pneumoconiosis by routine chest screening with plain films (Fig. 1a). He did not complain of cough, dyspnea, or other respiratory symptoms at the first visit to the hospital. On physical examination, the lung sounds were clear. Sputum cultures and AFB stain tests were performed to exclude tuberculosis; both were negative. Diagnostic computed tomography (CT) was performed and revealed numerous tiny scattered nodules and a few calcified nodules in both lungs. Multiple nodules showed peri-lymphatic distribution without enlarged lymph nodes (Fig. 1b). Wedge resection of the lung and a biopsy were performed for a definitive diagnosis and to identify the cause of the pneumoconiosis. On histologic examination, the specimen revealed mainly small airway-centered granulomatous micronodular lesions and multinucleated giant cells containing amorphous transparent particles (Fig. 2a and b). Polarizing microscopic findings of the same multinucleated giant cells shown in Fig. 2b reveals the birefringent particle (Fig. 2c). Transmission electron microscopic features highlight the intracytoplasmic electron lucent amorphous materials (asterisks) in the histiocytes (Fig. 2d). Scanning electron microscopic features and comparison element mapping of fluorine reveals the presence of fluorine elements in the pulmonary lesion. Scanning electron microscopic features of the lesion revealing fluorine elements show multiple round to oval granular material measuring 2–6 μm (Fig. 2e and f). The energy-dispersive X-ray spectroscopy (EDX) spectrum of a particle in a round particle found a multinucleated giant cell showing a prominent peak for fluorine (F) but with other associated elements such as carbon (C) and oxygen (O) (Fig. 2g and h). FT-IR was performed for elemental analysis of the lung tissue. The analysis showed vibration on a similar wavelength to the results of standard PTFE and PTFE spray solution, which had been used in the factory. This showed the presence of PTFE in the lung tissue **(**Fig. 3**)**.

#### Intervention and outcome

Six months after the first visit, the patient complained of chest pain and dyspnea. Since that time, he has been prescribed Singulair for symptomatic relief. He visited the outpatient clinic every year and underwent follow-up tests to follow the progress of the pneumoconiosis. He quit his job in September 2016.

### Occupational history

The work process at the workplace is summarized as follows. Round-shaped plates are processed into a frying-pan shape by a press machine. Surface sanding is then performed to increase the absorption rate of paint and coating material on the surface. After surface sanding, spray coating is performed. According to the material safety data sheet, the spray solution contains 55–65% PTFE. Subsequently, the pans are transported to a heat oven, and a drying process is performed. The coating process includes bottom, middle and top coating. Bottom coating is performed at 180 °C, while middle and top coating occurs at 400 °C. The dried pans are transported back through the spraying process until they are coated twice. Finally, the three layer-coated pans are assembled with handles.

The patient had been working for 28 years in only the PTFE spraying process. Spraying was performed 2000–3000 times a day, 50 cm away from the face. The patient worked 10 h a day, 6 days a week, without a respirator. The heat oven process for drying the pans was conducted approximately 1 m away from the spraying process; the heat oven is a long, closed structure with two small exits at opposite ends.

### Work environment

Fluorocarbon polymers, such as PTFE, are not target chemicals for routine work environment monitoring in Korea, so to identify exposure to PTFE, work environment air sampling was performed. First, qualitative analysis of air samples was conducted to confirm the presence of PTFE particles. Personal and regional samples were gathered from the workplace. A sample was taken from the breathing zone of the patient while working on the spraying process. A regional sample was taken from the top of the hood, approximately 2 m high, near the spraying process. Airborne sample collection was performed in accordance with the NIOSH Manual of Analytical Methods [9]. A cyclone and glass-fiber filter was used to collect the air samples. The flow rate was 1.6 L/min. The samples were collected for 6 h. For qualitative analysis of PTFE in the air samples, Fourier transform infrared spectroscopy (FT-IR) spectrometry was performed. The personal sample from the spray worker shows strong vibration at 1148.6 and 1204.9 cm^− 1^. FT-IR of a PTFE spray solution demonstrates peaks at 1117.7 and 1265.5 cm^− 1^. The spectra of the individual samples were consistent with the C-F bond characteristics of the sprays used at the factory. Scanning electron microscopy (SEM) analysis and EDX were then performed with the same samples to confirm the presence of PTFE particles and measure the size of the particles. Fine particles with a smooth, round surface showed fluorine and carbon peaks on the EDX spectrum. The particles found in the personal samples measured 1–22 μm by SEM; particles smaller than 1 μm were also found.

**Fig. 4:**
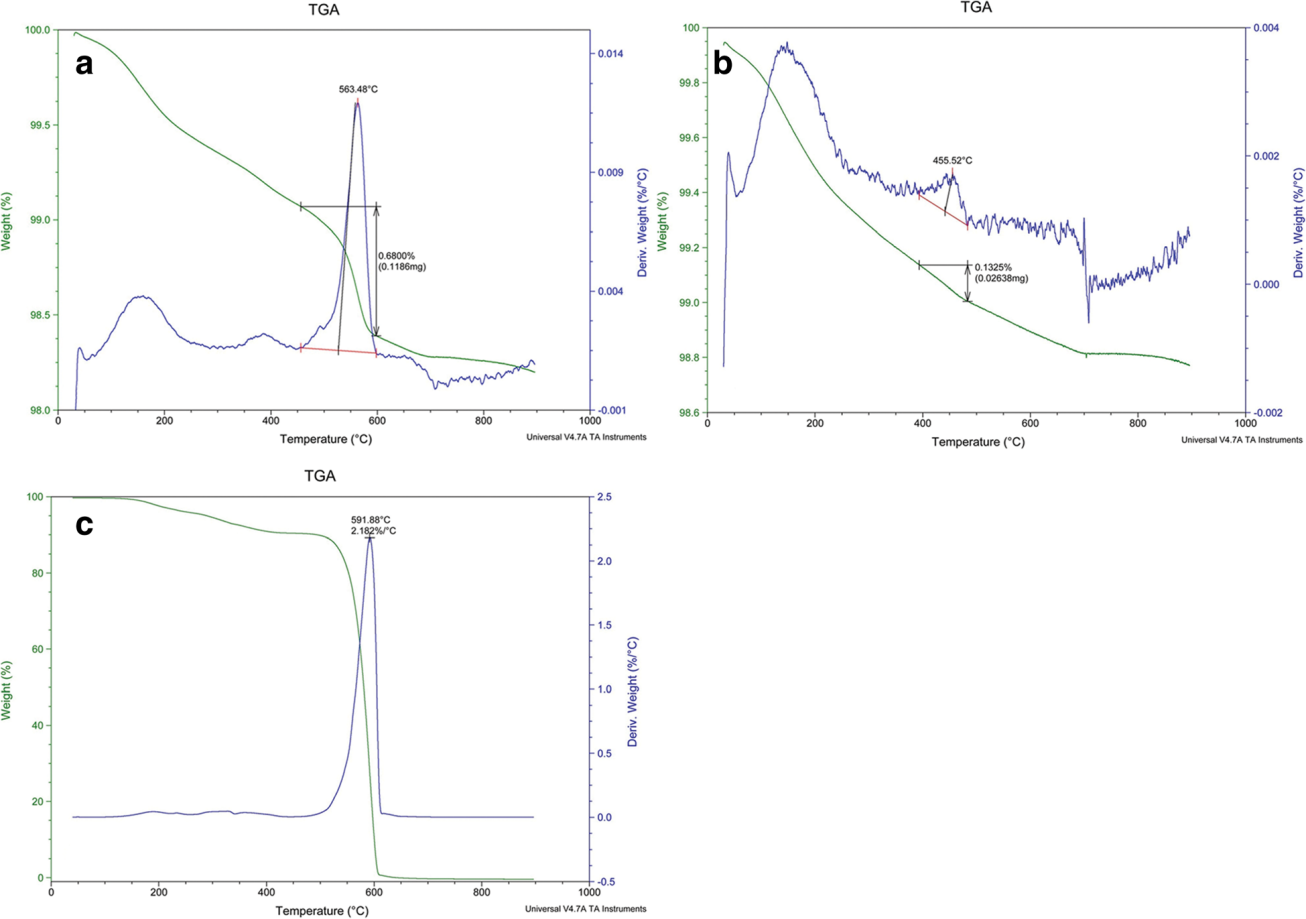
**a** Thermogravimetric analysis of the patient’s personal air sample shows prominent weight loss at 550–660 °C. A total of 0.40994 mg of the sample weight was reduced at 550–600 °C. **b** Thermogravimetric analysis of the regional sample shows weight loss near 450 °C, and no prominent weight loss was found at 550–600 °C. **c** Thermogravimetric analysis of the PTFE spray solution shows prominent weight loss at 550–600 °C

To conduct quantitative analysis of the particles, air samples were again collected. Personal and regional samples were gathered from the workplace. The personal sample was taken from the patient’s breathing zone using a cyclone sampler with a glass-fiber filter with a 1 μm pore size. The flow rate was 1.5 L/min, and the sample was collected for 6 h. A sample of 2.048 mg was taken. The regional sample was taken from the top of the hood using the cyclone sampler, and a PVC filter with a 5 μm pore size was used. The flow rate was 1.5 L/min, and the sample was taken for 6 h. A sample of 0.241 mg was taken. Thermogravimetric analysis (TGA) was then performed to differentiate the PTFE particles. A filter from the patient was divided into four, and TGA was performed. With the patient’s sample, prominent weight loss was shown at 550–600 °C. In total, 0.40994 mg of the sample weight was reduced at 550–600 °C **(**Fig. 4a**)*.*** The airborne concentration of PTFE was estimated to be 0.75 mg/m^3^. The regional sample showed no prominent weight loss at 550–600 °C **(**Fig. 4b**)*.*** To confirm that the weight loss point of the personal sample corresponded with PTFE, three types of PTFE spray solutions were used to coat the pans in the workplace for the top, middle, and primary coatings and were analyzed by TGA. All three solutions showed prominent weight loss at 550–600 °C, which corresponded with the results of the personal sample analysis **(**Fig. 4c**)**.

## Conclusion

This is a case report of small airway-centered granulomatosis caused by PTFE particles from the spraying process with a description of the air concentration of PTFE in a workplace. The patient was diagnosed with granulomatous lung disease from PTFE using CT and lung pathology and electron microscopic findings, which are compatible with a previously reported case [8]. We confirmed the presence of PTFE particles in the lung tissue by SEM and EDX of lung tissue. Additionally, the air sample from the workplace was analyzed by FT-IR, EDX, and TGA. The presence of PTFE was confirmed, and the diameter of the particles was measured. The air concentration was also calculated.

The patient’s CT findings showed numerous tiny scattered nodules and a few calcified nodules in both lungs; however, these were distinguishable from those of classic silicosis. The CT findings of typical silicosis include upper-lobe-dominant peri-lymphatic distribution of multiple 2–5 mm nodules with hilar and mediastinal lymph node enlargement and calcification [10]. Therefore, we ruled out silicosis as a diagnosis in this patient.

The respiratory effects of PTFE are usually focused on acute toxicity. Heated PTFE particles may cause symptoms that range from mild flu-like symptoms to severe symptoms, such as pulmonary edema [11, 12]. Various previous studies have suggested that ultrafine particles from the heating of PTFE severely injure the lungs, and the particles lose their toxicity after becoming coagulated into larger homogeneous particles [4, 13]. Acute pulmonary toxicity due to fluorocarbon-containing aerosol spray has been reported [14] from various work processes, such as those of waterproof leather, fabric spray, floor-stain protector, rust-proofing spray, grout sealer, and ski wax [15–18]. Choi et al. reported for the first time chronic pulmonary granulomatosis associated with exposure to PTFE [8]. The spraying process and aerosolized PTFE were excluded as the cause of small airway-centered granulomatosis because of the stability of PTFE in a liquid formulation [8]. However, nondegraded PTFE can induce an immunologic reaction in body tissue. PTFE has been used in various medical processes because it is well tolerated by the body tissue, not resorbed, and disperses in various fluids. However, foreign-body granulomatous reactions after the injection of PTFE have been reported, including Teflon granuloma formation after microvascular decompression [19], vocal cord injection for treating paralyzed vocal cords [20], suburethral injection for the treatment of vesicoureteral reflux in children [21], acetabular cup for hip replacement [22], and as a bulking agent for the treatment of stress urinary incontinence [23] has been reported. Foreign-body giant cell reaction and a glassy-appearing material in multinucleated giant cells are typical pathological findings of Teflon-induced foreign-body reaction [24]. Like these cases, multinucleated giant cells containing glassy-appearing material were frequently noted in the present case.

This patient worked on the same process for 28 years and never worked on other processes, including the heat-drying process. In our study, we collected both personal and regional samples from the workplace and analyzed them to identify the cause of small airway-centered granulomatosis. We confirmed the presence of PTFE particles of up to 20 μm by FT-IR, EDX, and TGA from the personal air samples. FT-IR, SEM and EDX analysis of the patient’s lung tissue showed the presence of 2–6 μm PTFE particles. The size of the pyrolyzed PTFE particles was 0.02–0.2 μm at 560 °C and 0.02–0.07 μm at 370 °C. Although pyrolyzed PTFE can aggregate into larger particle size, only particles pyrolyzed from high temperature up to 560 °C aggregated into large globular agglomerates, while particles pyrolyzed from 370 °C aggregated into chain shapes up to 1.6 μm in size [25]. In our study, the PTFE particles from air sampling measured 1–22 μm by electron microscopy, and the size corresponded to reported PTFE powder size (7.6 ± 8.5 μm) [26]. The particle size identified in the lungs was 2–6 μm. The coating process occurred at 180–400 °C; the size of pyrolyzed particles formed at this temperature would be smaller. Additionally, aggregated pyrolytic products of PTFE showed variety in shape such as spherical, undulating, concave, bowl or doughnut-shaped with a thickened peripheral portion [27]. In this study, the samples of PTFE collected from workplace revealed a round regular shape. Therefore, the particles are more likely to have originated from the spray process. As a result, we suggest that the small airway-centered granulomatosis diagnosed in this patient was caused by the aerosolized PTFE particles from the spraying process.

Patient lesions seemed to be caused by prolonged exposure to the aerosolized PTFE particles from the spraying process, without acute respiratory symptoms. One limitation of this study is that TGA was not performed on the collected lung tissue. Furthermore, the health effects of particles formed by pyrolysis cannot be excluded. When pyrolysis occurs, PTFE is decomposed into C_2_F_4_, C_3_F_6_, and C_4_F_8_ compounds [27]. We have not clearly excluded the presence of pyrolyzed PTFE particles such as CF_2_ = CF_2_, CF_3_ - CF = CF_2_ other than C-F bonds. In further studies, it will be necessary to quantitatively confirm pyrolyzed and non-pyrolyzed particles by separating samples according to particle size using an impactor and analyzing the samples via GC-MS. Epidemiological studies of chronic lung disease in workers using PTFE spray will also be needed.

In Korea, work environment monitoring is performed for metal dust, mineral dust containing silica, and several other dusts that are regulated by law. However, there is no regulation for measuring or controlling the concentration of many respirable particles, such as PTFE. Furthermore, no time-weighted average or short-term exposure limit is suggested for PTFE. Acute and chronic pulmonary diseases caused by PTFE have been reported, and further studies should be conducted to recognize toxicity and establish an exposure limit for PTFE. This study, with its quantitative analysis of the airborne concentration of PTFE, suggests a hazardous airborne concentration of PTFE and may support setting an exposure limit for PTFE.

## Abbreviations

CT: Computed tomography
EDX: Energy-dispersive X-ray spectroscopy
FT-IR: Fourier transform infrared spectroscopy
PTFE: Polytetrafluoroethylene
TGA: Thermogravimetric analysis
